# Rehabilitation of the Severely Atrophic Maxilla with Subperiosteal Implants: A Biomechanical and Decision Analysis of Material and Configuration Choices

**DOI:** 10.3390/biomimetics11060433

**Published:** 2026-06-18

**Authors:** Barış Erkut Türk, Bersu Bedirhandede, Dilan Gizem Doğan, Beyza Güney

**Affiliations:** 1 Department of Oral and Maxillofacial Surgery, Faculty of Dentistry, Gazi University, 06490 Ankara, Türkiye; 2 Department of Prosthodontics, Faculty of Dentistry, Gazi University, 06490 Ankara, Türkiye; bersubdede@gmail.com (B.B.); gizemdogan@gazi.edu.tr (D.G.D.); beyzabayat@gazi.edu.tr (B.G.)

**Keywords:** CFR-PEEK, finite element analysis, subperiosteal implant, titanium, TOPSIS

## Abstract

**Background/Objectives**: Patient-specific subperiosteal implants are increasingly used to treat severely atrophic ridges due to advances in digital planning and additive manufacturing. This study aimed to evaluate the effects of material type and implant configuration on stress distribution in subperiosteal implant systems and to compare their overall biomechanical performance using a multi-criteria decision framework. **Methods**: A three-dimensional model of a severely atrophic maxilla was reconstructed to simulate four clinical scenarios combining two configurations (one-piece and two-piece) and two materials (titanium and 60% carbon fiber-reinforced polyetheretherketone). Finite element analysis was conducted to assess stress distribution within the implant body, fixation screws, prosthetic framework, and surrounding bone under vertical and oblique loading conditions. Maximum and minimum principal stresses were evaluated in bone, whereas von Mises stresses were calculated for implant components. The resulting biomechanical indicators were subsequently integrated using an entropy weight–TOPSIS multi-criteria decision analysis. **Results**: Principal stresses in the surrounding bone showed minimal variation between titanium and 60% carbon fiber-reinforced polyetheretherketone across all configurations. Implant configuration had a more pronounced effect on implant body stress. Under oblique loading, the two-piece configuration demonstrated substantially higher implant stresses than the one-piece design, whereas under vertical loading, lower implant stresses were observed in the two-piece configuration. The multi-criteria analysis ranked the one-piece titanium model highest under oblique loading and the two-piece titanium model highest under vertical loading. **Conclusions**: Implant configuration and loading direction influenced biomechanical behavior more than material selection in patient-specific subperiosteal implants.

## 1. Introduction

Severe alveolar ridge atrophy presents a significant clinical challenge in implant dentistry, as the residual bone volume is often insufficient for the use of conventional endosseous implants. Management of such cases typically involves advanced surgical interventions, including sinus floor elevation, guided bone regeneration, pterygoid or zygomatic implant placement, inferior alveolar nerve lateralization, and distraction osteogenesis [1,2]. However, in the presence of extensive maxillary resorption, advanced sinus pneumatization, and compromised bone quality, these procedures require wide surgical access and are associated with considerable risks, including infection, graft failure, prolonged treatment duration, and increased cost [2].

Recent advances in additive manufacturing and computer-aided design/computer-aided manufacturing (CAD/CAM) technology have renewed clinical interest in patient-specific subperiosteal implants (SIs) [3]. Unlike endosseous implants, SIs are designed to rest on the cortical bone surface beneath the periosteum and are retained by fixation screws rather than osseointegration within the alveolar bone. This design principle makes SIs particularly suitable for patients with severely atrophic maxilla, as conventional bone augmentation or zygomatic implant placement would necessitate extensive surgical intervention in such cases [3,4]. SIs are fabricated based on individual anatomical morphology through digital imaging and additive manufacturing workflows. Preoperative computed tomography planning also enables precise identification of screw anchorage sites, thereby minimizing the risk of sinus membrane perforation and postoperative sinusitis [4]. The resulting framework typically consists of primary struts that form the main load-bearing structure and secondary struts arranged in a lattice-like configuration to reinforce overall rigidity and optimize load distribution across the cortical bone surface [5].

Material selection is a critical consideration in the design and long-term performance of SIs. Titanium (Ti) and its alloys remain the most widely used materials in dental implant manufacturing owing to their high mechanical strength, corrosion resistance, and established biocompatibility. Among Ti alloys, Ti-6Al-4V (Grade 5) offers a favorable balance between mechanical performance and corrosion resistance, making it well suited for load-bearing dental applications [1]. However, the vanadium content in this alloy has been associated with increased metal ion release and potential cytotoxic effects, raising concerns regarding long-term biological stability [6]. The high stiffness of Ti alloys may also contribute to stress shielding and subsequent bone resorption over time [1]. These limitations have motivated the investigation of alternative biomaterials with comparable mechanical properties and improved biological performance.

Polyetheretherketone (PEEK) has emerged as a promising alternative to metallic biomaterials, offering excellent chemical resistance, favorable biocompatibility, and a low elastic modulus that closely approximates that of cortical bone [1,7,8]. Its tooth- and bone-like color and the absence of corrosion-related metal ion release have further increased interest in its biomedical applications [9]. Reinforcement with carbon fibers further enhances its mechanical properties, producing carbon fiber-reinforced PEEK (CFR-PEEK), thereby bridging the stiffness gap between unreinforced PEEK and metallic implant materials [10,11].

Finite element analysis (FEA) has become an established computational tool for investigating the biomechanical behavior of dental implant systems. By enabling simulation of stress distributions within complex prosthetic and osseous structures under functional loading, FEA facilitates the optimization of implant geometry and material properties prior to clinical application [3]. The method allows identification of regions susceptible to mechanical failure and provides data that would be difficult to obtain through experimental methods alone [2].

Previous finite element studies have investigated the effects of SI material selection and design configuration on biomechanical performance [1,3,9,11]. Altıparmak et al. [10] evaluated the biomechanical response of Ti and 60% CFR-PEEK SI systems under vertical and oblique loading conditions. Ayhan and Cankaya [3] investigated stress distribution, displacement behavior, and bone loading characteristics of monoblock and dual custom-made SI designs in atrophic maxilla. Acar et al. [12] compared the biomechanical performance of subperiosteal and zygomatic implant configurations in unilateral atrophic maxillary defects subjected to different loading conditions. El Sawy et al. [1] further explored the effects of various subperiosteal and superstructure framework material combinations, including Ti, modified PEEK, and PEKK, on stress distribution patterns in atrophic maxilla using FEA. However, most of these investigations examined material and design variables independently, and the combined influence of material type and SI configuration on stress distribution has not been comprehensively evaluated. Moreover, conventional FEA studies typically compare stress values across individual components separately, without a systematic framework for integrating multiple biomechanical criteria into an overall performance assessment. Multi-criteria decision-making (MCDM) methods offer a structured approach to address this limitation [13]. Among these, the entropy weight-technique for order of preference by similarity to ideal solution (TOPSIS) method combines an objective weight-determination technique grounded in information theory with a distance-based ranking algorithm [14]. The entropy weight method derives criterion weights directly from the dispersion of the data, thereby eliminating subjective bias in importance assignment, while TOPSIS ranks alternatives according to their geometric proximity to the ideal and anti-ideal solutions across all criteria simultaneously. This combined approach has been increasingly adopted in engineering and biomedical applications for evaluating complex systems characterized by multiple conflicting performance criteria [15]. To the authors’ knowledge, no previous study has applied an integrated framework combining FEA and entropy-weighted TOPSIS to patient-specific SIs in a severely atrophic maxilla while simultaneously evaluating material choice, implant configuration, loading direction, and component-specific biomechanical responses. Accordingly, the present study aimed to analyze the stress distribution within the SI, fixation screws, prosthetic framework, and surrounding bone of Ti and 60% CFR-PEEK SI systems fabricated in one-piece and two-piece configurations under oblique and vertical loading conditions, and to evaluate the overall biomechanical performance using an entropy weight–TOPSIS multi-criteria decision analysis. The null hypothesis was that SI material, configuration, and loading direction would not significantly affect the stress distribution within the SI components and the surrounding bone.

## 2. Materials and Methods

### 2.1. Finite Element Analysis

**Figure 1:**
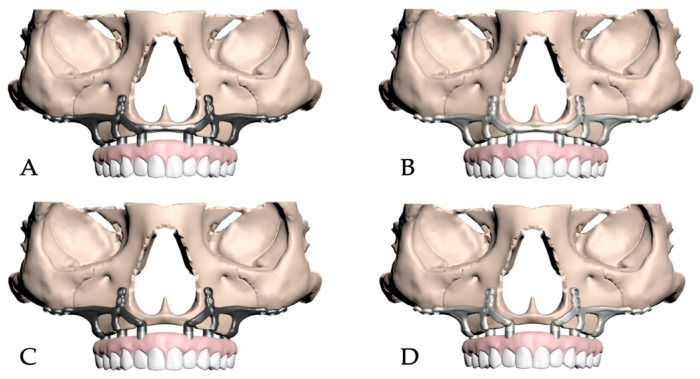
FEA models combining SI design and material. SI1-Ti (**A**); SI1-CFR-PEEK (**B**); SI2-Ti (**C**); SI2-CFR-PEEK (**D**).

The present FEA simulated a clinical scenario involving two SI designs: one-piece and two-piece, and two SI materials: Ti and 60% CFR-PEEK. A total of four models were constructed in the atrophic maxilla, each representing a different combination of material and design configuration (Figure 1):

- Model 1 (SI1-Ti): One-piece Ti SI
- Model 2 (SI2-Ti): Two-piece Ti SI
- Model 3 (SI1-CFR-PEEK): One-piece 60% CFR-PEEK SI
- Model 4 (SI2-CFR-PEEK): Two-piece 60% CFR-PEEK SI

The three-dimensional (3D) maxillary geometry was reconstructed using orthographic and cross-sectional images obtained from open-access digital anatomical sources [16,17,18]. These two-dimensional (2D) visual references guided the reconstruction of 3D geometries within a computer-aided design (CAD) environment, further validated according to Gray’s Anatomy for Students (42nd edition) and Netter’s Atlas of Human Anatomy (8th edition). The edentulous alveolar component was modified to simulate severe maxillary atrophy. Severe atrophy was defined as a residual ridge morphology insufficient for conventional endosseous implant placement without augmentation. The residual vertical distance from the nasal cavity floor to the alveolar crest was set at 4 mm, the crestal width was set at 3.5 mm, and cortical bone thickness was modeled as 1 mm [19,20]. These dimensions were selected to represent an advanced atrophic condition rather than an average edentulous maxilla, corresponding to a Cawood–Howell Class V/VI-type morphology characterized by deficient ridge height and width [21]. The one-piece and two-piece SI together with their respective abutment were modeled in Blender for Dental (New York, NY, USA), while fixation screws were designed in Solidworks (Dassault Systèmes SolidWorks Corp., Waltham, MA, USA). For the prosthetic component, tooth morphology was derived from Wheeler’s Dental Anatomy (11th edition). The SI was constructed with a thickness of 1.8 mm, and the fixation screws were modeled according to KLS Martin catalog specifications as MaxDrive screws with a diameter of 2 mm and a length of 7 mm. For modeling consistency, abutment emergence profiles were positioned 2 mm within the bone; gingival tissue was not included, as load transfer was evaluated at the implant–bone interface. After completing the design and assembly of all SI components, the conversion of the three-dimensional mesh structure into a mathematically appropriate solid model and the subsequent FEA were performed on a workstation equipped with an Intel Xeon CPU (3.4 GHz), 64 GB RAM, a 500 GB hard drive, and the Windows 10 Pro Version Service Pack 1 operating system. The models created in Blender for Dental and Solidworks were subsequently transferred to Simulia Abaqus software (Dassault Systèmes Simulia Corp., Johnston, RI, USA) while preserving the 3D coordinates. The model was meshed using 4-node linear tetrahedral elements (C3D4). A mesh convergence analysis was conducted, and mesh refinement was continued until the variation in von Mises equivalent stress between successive meshes was less than 5%, ensuring mesh-independent results. The total number of nodes and elements generated for the analysis is summarized in Table 1, and the material properties applied are presented in Table 2 [10,22]. Titanium and 60% CFR-PEEK were selected as SI materials, with elastic modulus values of 110 GPa and 150 GPa, respectively, according to previously reported data [10,23]. All materials were assumed to be homogeneous, isotropic, and linearly elastic.

Boundary conditions were defined by constraining the superior and posterior regions of the maxilla in all degrees of freedom to simulate skeletal fixation. Contacts between the subperiosteal implant and bone, as well as between fixation screws and surrounding bone, were defined as bonded [2,24,25].

Two distinct loading scenarios were simulated on the prosthetic framework. In the first protocol, vertical loads of 28 N, 94 N, and 108 N were applied to the first premolar, second premolar, and first molar regions, respectively. In the second protocol, an oblique load of 93 N was directed to the canine [22,26]. These loading conditions were selected to represent centric relation for maximum intercuspation and canine guidance for lateral occlusal forces, and the evaluation then examined the distribution of maximum principal stresses (Pmax) and minimum principal stresses (Pmin) within the surrounding bone, as well as von Mises stresses (VMS) generated in the SI, fixation screw, and prosthetic framework. Stress distribution patterns were visualized, where regions of higher stress appeared in red and lower stress in blue. Since FEA yields computational estimations rather than variable experimental data, no statistical evaluation was undertaken in this study.

### 2.2. Multi-Criteria Decision Analysis

To provide an objective, quantitative ranking of the investigated FEA models based on their overall biomechanical performance, a MCDM was performed using the entropy weight–TOPSIS method. The criteria for evaluating the FEA models are described in Table 3.

Separate decision matrices were constructed for each loading condition to evaluate performance under different clinical scenarios. To eliminate subjective bias in criterion importance assignment, entropy weighting was applied independently for each loading scenario in four steps:

Step 1. Proportional normalization was performed as:

$$p_{ij}=\frac{x_{ij}}{\sum_{i=1}^{m}x_{ij}}$$

where *i* represents the FEA model (*i* = 1,…, m), *j* is the criterion (*j* = 1,…, n), and x_*ij*_ is the evaluation of the FEA model *i* under decision criteria *j*.

Step 2. Entropy for each criterion was calculated as:

$$e_{j}=-k\sum_{i=1}^{m}p_{ij}ln(p_{ij})$$

with the normalization constant:

$$k=\frac{1}{ln(m)}$$

Step 3. The degree of diversification was defined as:

$$\;\;\;\;\;\;d_{j}=1-e_{j}$$

Step 4. The normalized entropy weight was obtained as:

$$w_{j}=\frac{d_{j}}{\sum_{j=1}^{n}d_{j}}$$

Criteria exhibiting greater dispersion among FEA models received higher weights, reflecting a stronger discriminative capacity within the given loading condition. Following entropy-derived weights, TOPSIS was applied in five steps:

Step 1. The decision matrix was normalized using Euclidean normalization as:

$$r_{ij}=\frac{x_{ij}}{\sqrt{\sum_{i=1}^{m}x_{ij}^{2}}}$$

Step 2. Each normalized value was multiplied by its corresponding entropy weight as:

$$v_{ij}=w_{j}\cdot r_{ij}$$

Step 3. Because all criteria were defined as cost-type attributes, the positive ideal solution (v^+^) was defined as the minimum value of each criterion, and the negative ideal solution (v^−^) as the maximum.
Step 4. The Euclidean distance of each alternative to the ideal best (D^+^_i_) and ideal worst (D^−^_i_) solutions was calculated as:

$$D_{i}^{+}=\sqrt{\sum_{j=1}^{n}(v_{ij}-v_{j}^{+}{)}^{2}}$$

$$D_{i}^{-}=\sqrt{\sum_{j=1}^{n}(v_{ij}-v_{j}^{-}{)}^{2}}$$

Step 5. The relative closeness coefficient was then determined as:

$$C_{i}=\frac{D_{i}^{-}}{D_{i}^{+}+D_{i}^{-}}$$

Higher C_*i*_ values indicate closer proximity to the ideal biomechanical condition. FEA model rankings were generated separately for oblique and vertical loading. To assess the sensitivity of the TOPSIS ranking to the weighting scheme, the analysis was repeated using equal weights (w_*j*_ = 0.20 for all criteria), thereby assigning identical importance to each biomechanical criterion regardless of its dispersion among the alternatives.

## 3. Results

### 3.1. Stress Distribution in Bone

**Figure 2:**
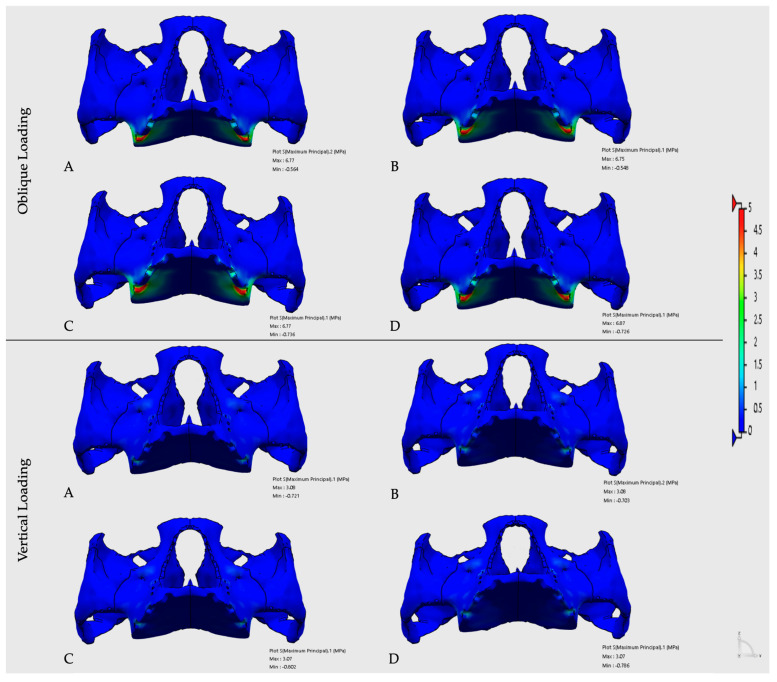
Maximum principal stress distributions in maxillary bone under vertical and oblique forces, expressed in MPa. SI1-Ti (**A**); SI1-CFR-PEEK (**B**); SI2-Ti (**C**); SI2-CFR-PEEK (**D**).

**Figure 3:**
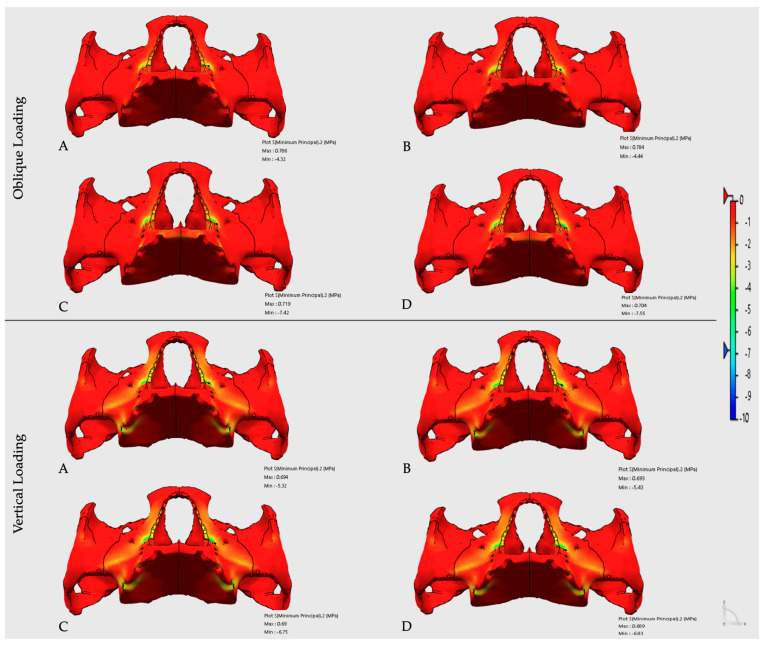
Minimum principal stress distributions in maxillary bone under vertical and oblique forces, expressed in MPa. SI1-Ti (**A**); SI1-CFR-PEEK (**B**); SI2-Ti (**C**); SI2-CFR-PEEK (**D**).

Pmax and Pmin values in the maxillary bone are summarized in Figure 2 and Figure 3. Pmax values were highest in the molar region in all models. In the one-piece configuration, Pmax values were 6.77 MPa under oblique loading and 3.08 MPa under vertical loading for Ti, and 6.75 MPa and 3.08 MPa for 60% CFR-PEEK, respectively. In the two-piece configuration, Pmax values were 6.77 MPa under oblique loading and 3.07 MPa under vertical loading for Ti, and 6.87 MPa and 3.07 MPa for 60% CFR-PEEK, respectively.

Pmin values were localized at the lateral aspect of the apertura piriformis in all models. In the one-piece configuration, Pmin values were −4.32 MPa under oblique loading and −5.32 MPa under vertical loading for Ti, and −4.44 MPa and −5.43 MPa for 60% CFR-PEEK, respectively. In the two-piece configuration, Pmin values were −7.42 MPa under oblique loading and −6.75 MPa under vertical loading for Ti, and −7.55 MPa and −6.83 MPa for 60% CFR-PEEK, respectively.

### 3.2. von Mises Stresses in Fixation Screws

**Figure 4:**
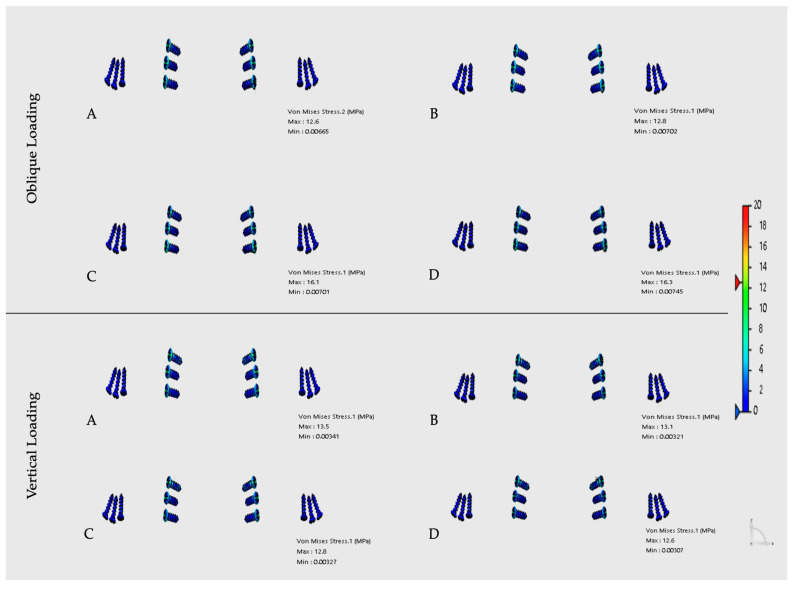
von Mises stresses in fixation screws under vertical and oblique forces, expressed in MPa. SI1-Ti (**A**); SI1-CFR-PEEK (**B**); SI2-Ti (**C**); SI2-CFR-PEEK (**D**).

Von Mises stress values in the fixation screws are presented in Figure 4. In the one-piece configuration, screw stresses were 12.6 MPa under oblique loading and 13.5 MPa under vertical loading for Ti, and 12.8 MPa and 13.1 MPa for 60% CFR-PEEK, respectively. In the two-piece configuration, screw stresses were 16.1 MPa under oblique loading and 12.8 MPa under vertical loading for Ti, and 16.3 MPa and 12.6 MPa for 60% CFR-PEEK, respectively.

### 3.3. von Mises Stresses in Prosthetic Frameworks

**Figure 5:**
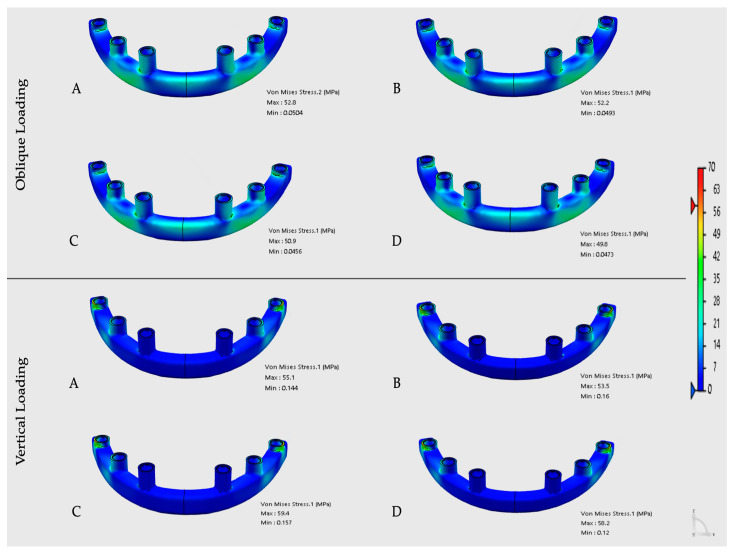
von Mises stresses in prosthetic frameworks under vertical and oblique forces, expressed in MPa. SI1-Ti (**A**); SI1-CFR-PEEK (**B**); SI2-Ti (**C**); SI2-CFR-PEEK (**D**).

Von Mises stress values in the metal frameworks are summarized in Figure 5. In the one-piece configuration, framework stresses were 52.8 MPa under oblique loading and 55.1 MPa under vertical loading for Ti, and 52.2 MPa and 53.5 MPa for 60% CFR-PEEK, respectively. In the two-piece configuration, framework stresses were 50.9 MPa under oblique loading and 59.4 MPa under vertical loading for Ti, and 49.8 MPa and 58.2 MPa for 60% CFR-PEEK, respectively. In all models, maximum stresses were observed in the canine or molar regions, whereas minimum values were located in the incisor region.

### 3.4. von Mises Stresses in Subperiosteal Implants

**Figure 6:**
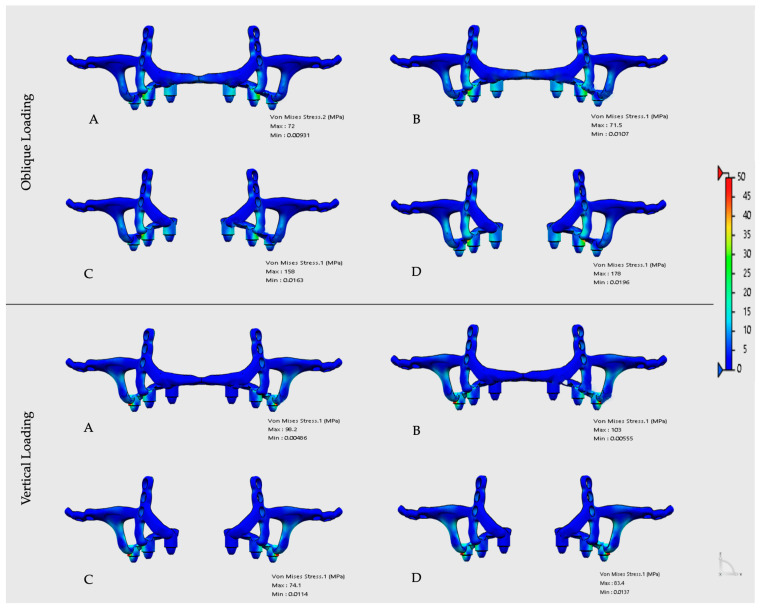
von Mises stresses in subperiosteal implants under vertical and oblique forces, expressed in MPa. SI1-Ti (**A**); SI1-CFR-PEEK (**B**); SI2-Ti (**C**); SI2-CFR-PEEK (**D**).

Von Mises stress values in the SIs are presented in Figure 6. In the one-piece configuration, SI stresses were 72.2 MPa under oblique loading and 98.2 MPa under vertical loading for Ti, and 71.5 MPa and 103 MPa for 60% CFR-PEEK, respectively. In the two-piece configuration, SI stresses were 158 MPa under oblique loading and 74 MPa under vertical loading for Ti, and 178 MPa and 83 MPa for 60% CFR-PEEK, respectively. In all models, maximum stresses were located in the canine or molar regions, whereas minimum values were observed in the incisor region.

### 3.5. Multi-Criteria Decision Analysis

The entropy-derived criterion weights for both loading conditions are summarized in Table 4. Under oblique loading, C5 received the highest weight (w = 0.6652), followed by C2 (w = 0.2743). C1 demonstrated the lowest weight (w = 0.0002). Under vertical loading, C5 received the highest weight (w = 0.5120), followed by C2 (w = 0.4143).

The TOPSIS closeness coefficients and corresponding rankings for both loading conditions are presented in Table 5. Under oblique loading with entropy weights, SI1-Ti achieved the highest closeness coefficient (C_*i*_ = 0.9955), followed by SI1-CFR-PEEK (C_*i*_ = 0.9905). One-piece configurations demonstrated higher closeness coefficients than two-piece configurations under this loading condition. Under vertical loading with entropy weights, the ranking was reversed, and SI2-Ti ranked first (C_*i*_ = 0.6291), followed by SI2-CFR-PEEK (C_*i*_ = 0.4906).

## 4. Discussion

The present study compared the effects of different SI materials and design configurations on stress distribution within the SI, fixation screws, prosthetic framework, and surrounding bone under both vertical and oblique loading conditions, and the results demonstrated that SI design, material selection, and loading direction influenced biomechanical behavior.

In the present study, the loading protocol simulated centric intercuspation with 28 N, 94 N, and 108 N vertical loads applied to the first premolar, second premolar, and first molar regions, respectively, and canine-guided occlusion with a 93 N oblique load on the canine region [22,26]. These values are within the reported physiological chewing force range of 300–550 N [27,28], which supports the clinical relevance of the chosen parameters. Canine-guided occlusion was selected as it is considered biomechanically more favorable for implant-supported restorations, mainly because it distributes lateral forces more effectively, as also reported by Sentürk and Akaltan [29] and Bozyel et al. [30]. Since masticatory forces act in both vertical and oblique directions, including oblique loading in the FEA model was also important to obtain more realistic stress distribution patterns [10].

Beyond the applied loading conditions, the interpretation of the present FEA results also depends on the contact assumptions governing load transfer. From a methodological perspective, an important consideration is the use of bonded interfaces between the SI and cortical bone, as well as between the fixation screws and surrounding bone. This bonded definition was not intended to represent conventional biological osseointegration of the SI, since contemporary SIs are positioned on the cortical bone surface and achieve stability primarily through anatomical adaptation and fixation screws rather than endosseous osseointegration [25,31]. Instead, the bonded condition was used to represent complete seating of the SI on the cortical maxillary surface and stable fixation screw engagement under loading. Similar idealized contact assumptions have been adopted in previous SI finite element studies. Demir and Caglar [24] modeled the framework–maxillary bone interface as bonded to simulate the extensive contact area and fibrointegration contributing to SI stabilization, whereas Canko and Doganay Ozyilmaz [25] defined the implant-related contacting components as bonded. However, this assumption may overestimate interface stability while underestimating local stress concentrations and displacement under clinical conditions. In the presence of frictional contact, imperfect framework adaptation, local interfacial gaps, or micromotion, load transfer may not occur uniformly across the entire cortical contact surface. Instead, stresses may become concentrated around fixation screws, screw holes, and localized cortical support regions. Carnicero et al. [32] demonstrated that incorporation of screw–bone interaction in SI structures significantly influences displacement behavior and reaction forces, and that cortical stress distribution is strongly affected by screw thread–bone interaction. Similarly, De Moor et al. [33] emphasized the biomechanical relevance of bone–SI relative motion, reporting that limited micromotion may contribute to bone ingrowth and secondary stabilization. Therefore, the bonded interface assumption in the present study reflects an idealized mechanically stabilized condition.

To evaluate the biomechanical response under these loading conditions, von Mises stress was used for the SI, fixation screws, and prosthetic framework, whereas maximum and minimum principal stresses were calculated for the supporting bone. This approach is consistent with established biomechanical practice, as von Mises criteria are appropriate for ductile metallic structures, while principal stresses better reflect tensile and compressive responses in brittle materials [6,7].

Based on the principal stress analysis, the influence of implant material on surrounding bone stress was minimal. Despite a 36% difference in elastic modulus between Ti and 60% CFR-PEEK, maximum variations in principal stresses did not exceed 0.10 MPa for Pmax and 0.13 MPa for Pmin across all design–loading conditions. These findings are in line with previous finite element studies. Schwitalla et al. [34] reported nearly identical cortical bone stress for Ti and 60% CFR-PEEK implants, while low-modulus PEEK (4 GPa) showed distinctly different stress patterns. Zhou et al. [23] also found comparable stress and strain distributions for Ti and 60% CFR-PEEK across various bone density types, and Pérez-Pevida et al. [35] observed similar bone stress values for implant materials with elastic moduli ranging from 67 to 210 GPa. Korabi et al. [36] further confirmed through parametric modeling that bone failure criteria remain stable once implant stiffness exceeds that of cortical bone. These findings suggest that once implant stiffness exceeds the cortical bone range, further increases in rigidity have little effect on surrounding bone stress.

This limited material influence may be further reinforced by the macrogeometry of subperiosteal implants. Unlike threaded endosseous implants, which concentrate load transfer at a localized bone–implant interface, SIs distribute occlusal forces over a broad cortical surface through multiple fixation points. This plate-like load distribution reduces stress concentration and further diminishes the relative contribution of material stiffness to surrounding implant bone stress.

Material-related differences were more evident in the SI than in the surrounding bone. In most configurations, the 60% CFR-PEEK exhibited higher VMS within the SI compared with Ti. This finding is consistent with fundamental mechanical principles, as a material with a higher elastic modulus develops greater internal stress when subjected to the same level of strain under functional loading [37]. Pérez-Pevida et al. [35] similarly demonstrated that implant stress increases proportionally with elastic modulus across a wide stiffness range, while surrounding bone stress remains stable. In contrast, Altıparmak et al. [10] reported higher von Mises stress in titanium compared with 60% CFR-PEEK SI. Given the comparable material properties and loading magnitudes in both studies, this discrepancy most likely reflects differences in geometric design, including framework extension, fixation screw distribution, and abutment configuration, which influence load pathways and bending behavior.

Increased VMS within the 60% CFR-PEEK SI was accompanied by a slight reduction in stress within the prosthetic framework. As bone stress was comparable between materials, this pattern indicates that the additional load carried by the stiffer implant was redistributed within the prosthetic assembly rather than transferred to the supporting bone. A similar trend was reported by El-Sawy et al. [1], showing that stiffer SI retained more internal stress and transferred less load to bone and fixation screws.

Stress distribution within the SI was also affected by implant configuration, with distinct patterns observed under oblique and vertical loading. Under oblique loading, the two-piece design generated higher SI stress than the one-piece configuration, with VMS values exceeding twice those of the one-piece design. This pattern suggests that interruption of cross-arch structural continuity amplifies bending effects when lateral force components are present, resulting in localized stress concentration within the segmented framework.

Under vertical loading, the design-related response differed. SI VMS values were comparable between configurations and, in certain conditions, slightly higher in the one-piece design. A similar load direction-dependent variation was reported by Arı and Acar [38], with application of 28 N, 94 N, and 108 N vertical loads to the first premolar, second premolar, and first molar, respectively, and a 93 N oblique load to the canine, resulting in stress magnitudes varying between structural components according to force orientation. Likewise, Ayhan and Cankaya [3] observed reduced implant stress in two-piece configurations under simulated vertical masticatory loads, indicating that segmentation may modify internal load transfer pathways when forces act primarily along the implant axis. The present findings partially align with this observation under vertical loading but diverge under oblique loading, indicating that the biomechanical advantage of one-piece or two-piece configurations cannot be generalized without considering the direction of the applied load. This is consistent with the broader principle that framework geometry significantly influences internal stress distribution in customized SIs, as demonstrated by Carnicero et al. [32] and Ronsivalle et al. [39], who showed that structural configuration alters stress concentration patterns and load transfer pathways.

This load-direction dependency was not confined to the SI structure but was also reflected in the supporting bone. Although the vertical load was higher in magnitude, oblique loading tended to generate higher principal stress values in cortical bone. This finding is consistent with basic mechanical behavior. Vertical forces are mainly transferred along the implant axis and produce predominantly compressive stress. In contrast, oblique forces introduce a horizontal component that generates bending and shear, leading to higher tensile and compressive gradients within cortical bone [2,34,40]. Previous finite element studies have similarly reported that non-axial loading results in greater stress concentration in surrounding bone. Schwitalla et al. [34] demonstrated increased stress under oblique forces compared with vertical forces. Higher principal stress values in specific bone regions under oblique loading have also been reported in SI systems subjected to similar loading conditions [12,38]. Gomes et al. [40] further showed that canine-guided loading produces higher horizontal strain compared with more distributed occlusal patterns.

To consolidate the biomechanical findings across multiple stress criteria simultaneously, an entropy weight-TOPSIS multi-criteria decision analysis was performed. The entropy-derived weights identified implant body stress as the most discriminative criterion under both loading conditions, whereas maximum principal bone stress contributed minimally to the differentiation among configurations, consistent with the negligible material-related variation observed in tensile bone stress. Since entropy-derived weights reflect the statistical dispersion of criterion values among the tested configurations rather than their direct clinical importance, the near-zero weight assigned to maximum principal stress should be interpreted as limited discriminative capacity among the models rather than clinical irrelevance. Accordingly, the entropy weight-TOPSIS results should be considered a data-driven decision-support ranking based on the selected biomechanical criteria rather than a definitive indicator of clinical superiority. This interpretation is consistent with a previous report indicating that entropy weighting amplifies the influence of highly variable criteria rather than their actual clinical significance [41]. Furthermore, sensitivity analysis using equal weights produced consistent ranking patterns, with one-piece configurations maintaining superior performance under oblique loading and SI2-Ti retaining the first rank under vertical loading, confirming that the principal findings were not dependent on the weighting strategy.

The TOPSIS ranking exhibited a pronounced load-direction dependency. Under oblique loading, both one-piece configurations were ranked substantially higher than both two-piece configurations, whereas under vertical loading, this hierarchy was reversed. This finding corroborates the present FEA and further supports the premise that the biomechanical superiority of a given design configuration cannot be generalized independently of loading direction. Moreover, variations in design configuration produced considerably larger shifts in closeness coefficients than variations in material within the same design category, suggesting that structural continuity and framework geometry exert a greater influence on overall biomechanical performance than material selection. This observation is in agreement with the finding that, once implant stiffness exceeds the cortical bone range, the macrogeometric characteristics of the implant framework become the predominant factor governing system-level stress distribution in SI systems.

From a clinical perspective, the findings suggest that implant configuration may need to be considered together with the dominant loading pattern rather than material stiffness alone. In patients with pronounced lateral excursions or parafunctional activity, oblique force components may impose mechanically critical bending loads on the framework. Under such conditions, maintaining cross-arch structural continuity through a one-piece configuration may reduce implant body stress concentration and improve mechanical stability. Conversely, in cases where posterior axial loading predominates and lateral components are limited, a two-piece configuration may reduce implant body stress by modifying internal load transfer pathways. However, these findings should be interpreted with caution, as simplified boundary conditions and the absence of soft tissue and muscle forces may have influenced the simulated stress distribution patterns and limited the physiological realism of the model [42].

The present study has several limitations. All materials, including bone and implant components, were assumed to be homogeneous, isotropic, and linearly elastic, although their actual mechanical behavior may be heterogeneous and anisotropic [10,43]. These simplifications, while necessary for modeling, may affect the accuracy of stress distribution. Most FEA studies, including the present one, also presume complete osseointegration and exclude muscle forces and bone remodeling processes. In addition, the lack of a standardized FEA modeling protocol in dentistry complicates comparison among studies, and simplifications in load and material definitions may not fully reflect clinical conditions. The applied loading forces were based on reference ranges that can vary depending on individual factors such as age, sex, and opposing dentition. The selected vertical and oblique loading protocols enabled standardized comparison among the SI models; however, they cannot fully reproduce functional mastication, which involves multidirectional cyclic loading and substantial patient-specific variability [44,45]. Occlusal loading may vary according to force magnitude and direction, occlusal scheme, opposing dentition, parafunctional habits, prosthetic design, and patient-specific factors [26,45]. Therefore, the present findings should be interpreted as static responses under selected loading scenarios rather than as a complete representation of time-dependent functional behavior. Repeated cyclic loading may influence fatigue behavior, prosthetic component stability, and peri-implant bone response over time [44,46,47]. Finally, since FEA is a deterministic method relying on fixed boundary conditions, it cannot account for biological variability, which limits the clinical generalization of the results [2]. Future FEAs should evaluate frictional behavior, adaptation gaps, and micromovement to better approximate clinical conditions, while future in vivo and clinical investigations are needed to validate the present findings and clarify the long-term biomechanical performance of SI configurations under functional loading.

## 5. Conclusions

Within the limitations of this finite element analysis, implant configuration and loading direction had a greater influence on the biomechanical behavior of SI systems than material substitution. Although Ti and 60% CFR-PEEK showed similar stress values in the surrounding bone, structural configuration significantly affected stress distribution within the implant body depending on the loading direction. One-piece designs demonstrated lower stresses under oblique loading, whereas two-piece configurations showed lower SI stresses under vertical loading. These findings highlight the importance of considering both implant design and loading direction in the biomechanical evaluation of patient-specific SIs. Optimizing implant configuration according to expected functional loading patterns may contribute to improved mechanical performance and long-term stability of customized implant frameworks. However, these findings should be interpreted within the limitations of the present finite element model, and further in vitro dynamic/fatigue testing, experimental and clinical validation are needed.

## Figures and Tables

**Table 1:** Mesh density of the SI designs.

	Number of Nodes	Number of Elements
One-piece SI	651,536	2,809,641
Two-piece SI	359,287	1,516,734

**Table 2:** Material properties used in the FEA.

Materials	Modulus of Elasticity (MPa)	Poisson’s Ratio
Cortical Bone	13,700	0.30
Spongious Bone	1370	0.30
Prosthetic Framework (Co-Cr)	218,000	0.33
Polymethyl methacrylate	3000	0.35
Titanium	110,000	0.34
60% CFR-PEEK	150,000	0.356

**Table 3:** Description of biomechanical performance criteria for evaluating FEA models.

Criteria No.	Criteria	Brief Description of Criteria	Implications on Biomechanical Performance
C1	Pmax (MPa)	Maximum principal (tensile) stress in supporting bone; tensile stress can initiate micro-cracks in cortical bone	Lower-the-better
C2	Pmin (MPa)	Minimum principal (compressive) stress magnitude in bone; excessive compressive stress causes bone resorption	Lower-the-better
C3	Fixation screw VMS (MPa)	Von Mises stress in fixation screws; high stress leads to screw loosening or fatigue fracture	Lower-the-better
C4	Prosthetic framework VMS (MPa)	Von Mises stress in the prosthetic framework (bar); framework fracture requires prosthesis replacement	Lower-the-better
C5	SI VMS (MPa)	Von Mises stress in the implant body; implant fracture is a catastrophic failure requiring surgery	Lower-the-better

**Table 4:** Entropy values, divergence coefficients, and entropy-derived criterion weights for oblique and vertical loading conditions.

	Oblique Loading			Vertical Loading		
Criteria	*Ej*	*dj*	*wj*	*Ej*	*dj*	*wj*
C1	1.0000	0.0000	0.0002	1.0000	0.0000	0.0001
C2	0.9750	0.0250	0.2743	0.9951	0.0049	0.4143
C3	0.9947	0.0053	0.0583	0.9998	0.0002	0.0206
C4	0.9998	0.0002	0.0020	0.9994	0.0006	0.0530
C5	0.9393	0.0607	0.6652	0.9939	0.0061	0.5120

**Table 5:** TOPSIS closeness coefficients (C_*i*_) and rankings under entropy-derived and equal weights for oblique and vertical loading conditions.

	Oblique Loading (Entropy)		Oblique Loading (Equal)		Vertical Loading (Entropy)		Vertical Loading (Equal)	
	*C_i_*	*Rank*	*C_i_*	*Rank*	C_i_	*Rank*	*C_i_*	*Rank*
SI1-Ti	0.9955	1	0.9451	2	0.4355	3	0.4866	2
SI1-CFR-PEEK	0.9905	2	0.9500	1	0.3659	4	0.4389	4
SI2-Ti	0.1810	3	0.1563	3	0.6291	1	0.5596	1
SI2-CFR-PEEK	0.0002	4	0.0546	4	0.4906	2	0.4493	3

## Data Availability

The original contributions presented in this study are included in the article. Further inquiries can be directed to the corresponding author.

## Abbreviations

The following abbreviations are used in this manuscript:

CAD/CAM	Computer-aided design/computer-aided manufacturing
SI	Subperiosteal implants
Ti	Titanium
PEEK	Polyetheretherketone
CFR-PEEK	Carbon fiber-reinforced polyetheretherketone
FEA	Finite Element Analysis
MCDM	Multi-criteria decision-making
TOPSIS	Technique for order of preference by similarity to ideal solution
2D	Two-dimensional
3D	Three-dimensional
Pmax	Maximum Principal Stress
Pmin	Minimum Principal Stress
VMS	von Mises Stress
